# The C-Terminal Domain of RNase H and the C-Terminus Amino Acid Residue Regulate Virus Release and Autoprocessing of a Defective HIV-1 Possessing M50I and V151I Changes in Integrase

**DOI:** 10.3390/v14122687

**Published:** 2022-11-30

**Authors:** Tomozumi Imamichi, Qian Chen, Ming Hao, Weizhong Chang, Jun Yang

**Affiliations:** Laboratory of Human Retrovirology and Immunoinformatics, Applied and Developmental Directorate, Frederick National Laboratory, Frederick, MD 21702, USA

**Keywords:** HIV-1, integrase, RNase H, virus release, autoprocessing, maturation

## Abstract

Previously, we reported that an HIV-1 variant containing Met-to-Ile change at codon 50 and Val-to-Ile mutation at codon 151 of integrase (IN), HIV(IN:M50I/V151I), was an impaired virus. Despite the mutations being in IN, the virus release was significantly suppressed (*p* < 0.0001) and the initiation of autoprocessing was inhibited; the mechanism of the defect remains unknown. In the current study, we attempted to identify the critical domains or amino acid (aa) residue(s) that promote defects in HIV(IN:M50I/V151I), using a series of variants, including truncated or aa-substituted RNase H (RH) or IN. The results demonstrated that virus release and the initiation of autoprocessing were regulated by the C-terminal domains (CTDs) of RH and IN. Further studies illustrated that Asp at codon 109 of RH CTD and Asp at the C terminus of IN induces the defect. This result indicated that the CTDs of RH and IN in GagPol and particular aa positions in RH and IN regulated the virus release and the initiation of autoprocessing, and these sites could be potential targets for the development of new therapies.

## 1. Introduction

Nascent viral particles of human immunodeficiency virus type-1 (HIV-1) released from HIV-1-producing cells are immature and non-infectious [1,2]. The particles contain Gag and GagPol polyproteins, regulatory viral proteins (Vif, Vpr, Vpu, Tat, Rev, and Nef), and two molecules of single-stranded genomic RNAs with host proteins [2,3,4]. Gag and GagPol polyproteins and the regulatory viral proteins are encoded in a single genomic RNA. GagPol polyproteins are produced by a −1 nucleotide (nt) ribosomal frameshifting event during the translation of the Gag protein [5]. A ratio of translated Gag to GagPol is generally known as 20:1 in HIV-1-producing cells [6]. The maintenance of the ratio is important for HIV infectivity [7]. Gag and GagPol target the plasma membrane via the myristoylated Gly of matrix protein (MA) at the N terminus of the polyprotein. Gag consists of MA, capsid (CA), nucleocapsid (NC), the spacer peptides p2 and p1, and p6 domains; GagPol comprises MA, CA, NC, p6*, p2, p1, protease (PR), reverse transcriptase (RT), RNase H (RH), and integrase (IN) [1]. Each domain is concatenated via the sequences of PR cleavage site to form the polyprotein chains [1]. Gag and GagPol assemble into a hexagonal lattice via its CA domain, recruiting viral proteins and the genomic viral RNAs. During the assembly process, Gag and GagPol translocated to the membrane form buds with the curved Gag lattices on the cell surface. These structures are formed corresponding to the bending of the membrane at the site, followed by the recruitment of the endosomal sorting complex III (ESCRT-III) components required for transport by the p6 domain of Gag, and induce membrane scission and the release of nascent virus particles [2,8,9,10,11].

The nascent immature HIV-1 particles mature into infectious viruses through a sequential processing step of the enzymatic cleavage of Gag and GagPol proteins by the mature PR [2,3]. The newly translated, embedded PR in the GagPol polyprotein is not a fully functional enzyme. However, it is converted into a mature functional enzyme via self-cleavage in an intramolecular manner (*cis*) [12]; this step is called autoprocessing. Once the protein is converted to its active form, it cleaves the Gag and GagPol polyproteins at the cleavage sites and scissors each functional protein inside the virus particle during budding or after releasing [1]. The initiation of the autoprocessing step is primed at the junction between p2 and NC [12,13,14]. This initial processing step is caused via an intramolecular digestion (the embedded PR cleaves the polyprotein at the initial cleavage site on the same polyprotein chain) [12]. The first digestion triggers a cascade of enzymatic reactions to release the functional mature PR. The order of the cleavage sequence is a strict regulation [12,13,14]. The autoprocessing step is suppressed by PR inhibitors [15,16] or regulated by truncated IN [17,18,19,20], amino acid substituted IN [21,22] or RH [22]; thus, the autoprocessing step is considered a target for developing novel anti-HIV drugs.

A genome-wide association study of HIV genomic RNA demonstrated that a total of 14 non-synonymous single nucleotide polymorphisms (SNPs) were correlated with plasma viral load (VL) in anti-retrovirus treatment-naive HIV-infected patients [23]. The mutations were located on CA, RH, IN, envelope, and Nef. We previously investigated the roles of each SNP in viral fitness in vitro using a laboratory-adapted HIV clone, HIV_NL4.3_, as a parent strain, and site-directed mutagenesis was conducted to induce individual or a combination of the SNPs. We found that a mutant containing a Met-to-Ile change at codon 50 of IN (IN:M50I) was impaired. The mutant suppressed the virus release to 0.3% of wild-type HIV_NL4.3_ (WT) and inhibited the GagPol autoprocessing step. These results indicated that IN regulates virus release and autoprocessing. Other groups reported that IN regulated maturation/processing using IN-deleted/truncated HIV mutants [17,18,19,21]. Therefore, our finding using aa-substituted IN consists of the previous reports. The defect was rescued by the other co-existing VL-associated SNP—Ser-to-Asn change at codon 17 of IN (IN:S17N) [22]. Interestingly, an Asn-to-Ser change at codon 79 of RH (RH:N79S) also restored virus release, autoprocessing, and replication competence of the defective IN:M50I virus [22]. These results indicated that RH is also involved in virus release and autoprocessing, thereby affecting virus fitness.

We investigated a population of HIV variants containing the IN:M50I mutation in patients participating in NIAID clinical trials. Surprisingly, the plasma VL level in patients infected with HIV containing IN:M50I without the compensatory mutations (either IN:S17N or RH:N79S) was 1207–201,353 copies/mL [24], suggesting that the clinical isolates containing IN:M50I were not impaired in the virus released from HIV-producing cells, unlike our previous in vitro study. To clarify this discrepancy between the in vitro and in vivo data, we performed a comparative analysis of the RNA genome of the entire *pol* region. We then found that the recombinant HIV(IN:M50I) virus contained a Val-to-Ile mutation at codon 151 of IN (IN:V151I) in the backbone [24]. IN:V151I is an endogenous aa substitution in HIV_NL4.3_ [25], and is known as a polymorphic mutation associated with drug resistance [26,27,28,29,30]. Given the reports that a recombinant consensus B HIV_NL4.3_ IN protein-containing M50I and V151I is functional [31], while our recombinant virus possessing the mutations was defective in virus release and autoprocessing, we hypothesized that backbone mutations in the HIV(M50I/V151I) may regulate virus release, autoprocessing, and replication activities in in vitro studies.

In the current study, we further investigated the mechanism of the defects in virus release and autoprocessing in HIV(M50I/V151I) and reported that the C-terminal domains of RH and IN are associated with the deficiency. These results provide a potential target for the development of anti-HIV drugs/therapy.

## 2. Materials and Methods

### 2.1. Ethics Statement

Approval for these studies, including all sample materials and protocols, was granted by the National Institute of Allergy and Infectious Diseases (NIAID) Institutional Review Board, and participants were provided the informed written consent prior to blood being drawn. All experimental procedures in these studies were approved by the National Cancer Institute at Frederick and Frederick National Laboratory for Cancer Research (the protocol code number: 16–19, approval date: 6 January 2017).

### 2.2. Cells

Peripheral blood mononuclear cells (PBMCs) were isolated from healthy donors’ apheresis packs (NIH blood bank) using a lymphocyte separation medium (ICN Biomedical, Aurora, OH, USA) [22,24]; CD4(+) T cells were purified from PBMCs using CD4 MicroBeads (Miltenyi Biotec, Auburn, CA, USA), according to the manufacturer’s instructions. The purity of the cell types was at least 90%, based on the flow cytometric analysis. Cell viability was determined using the trypan blue (Thermo Fisher, Waltham, MA, USA) exclusion method. HEK293T cells were obtained from ATCC (Manassas, VA, USA) and maintained in complete D-MEM (Thermo Fisher) supplemented with 10 mM 4-(2-hydroxyethyl)-1-piperazineethanesulfonic acid (HEPES), pH 7.4 (Quality Biological, Gaithersburg, MD, USA), 10% (*v*/*v*) fetal bovine serum (FBS; Thermo Fisher), and 50 μg/mL gentamicin (Thermo Fisher), as previously described [32].

### 2.3. Construction of Plasmids Encoding HIV Variants

Plasmids encoding HIV variants were constructed by site-directed mutagenesis on pNL4.3 [33] (the plasmid was obtained from M. Martin through the AIDS Research and Reference Reagent Program, National Institute of Allergy and Infectious Diseases, National Institutes of Health). Site-directed mutagenesis was performed using a QuickChange Lightning kit (Agilent Technologies, Santa Clara, CA, USA) with mutagenesis primers (Table S1), as previously described [22]. RH- or IN-deleted constructs were created by inverting PCR using Pfu II Taq polymerase (Agilent Technology) with deletion primers (Table S1). Mutagenesis was confirmed by Sanger DNA sequencing using the BigDye terminator v.3 (Thermo Fisher) with SeqStudio Genetic Analyzer (Thermo Fisher), and the plasmids were maintained in Stbl3 *E. coli* (Thermo Fisher). The plasmid purification was carried out using the EndoFree Plasmid Maxi Kit (Qiagen, Germantown, MD, USA).

### 2.4. Recombinant HIV-1 Viruses

Recombinant HIV-1 variants were prepared by transfecting the pNL4.3 mutants into HEK293T cells using TransIT-293 (Mirus, Houston, TX, USA) and Opti-MEM I medium (Thermo Fisher) following a method previously reported [22]. Culture supernatants were collected at 48 h after transfection and then filtrated through 0.45 μm pore-size filter membranes (MiliporeSigma, Burlington, MA, USA). Virus particles in the filtrate were pelleted by ultra-centrifugation on 20% (*w*/*v*) sucrose (MiliporeSigma) in 10 mM HEPES-150 mM NaCl buffer (pH 7.4), as previously described [22,34]. The pelleted virus particles were washed with PBS and then resuspended in PBS in a 1/100 vol of supernatants. The virus stocks were stored at −80 °C until use. The concentration of HIV p24 antigen in each stock was determined by a p24 antigen capture kit (PerkinElmer, Waltham, MA, USA) and the concentration of total virus proteins were determined by a BCA protein assay kit (Thermo Fisher) [32,34].

### 2.5. HIV Replication Assay

The levels of HIV replication of each variant were determined using primary CD4(+) T cells as previously described [22]. CD4(+) T cells were stimulated with 5 μg/mL phytohemagglutinin (PHA, MiliporeSigma) in complete RPMI-1640 (Thermo Fisher) supplemented with 10 mM HEPES, 10% (*v*/*v*) FBS, and 50 μg/mL gentamicin (RP10). The PHA-stimulated CD4(+) T cells (10 × 10^6^ cells) were infected with 10 ng of p24 of each HIV variant at 10 × 10^6^ cells/mL in RP10 for two hours at 37 °C. The infected cells were washed with RP10, and then cultured at 1 × 10^6^ cells/mL in the medium in the presence of 20 units/mL of recombinant IL-2 (MiliporeSigma) for 14 days at 37 °C in T25 flasks [22,24]. Half of the cell-free culture supernatants were exchanged with fresh RP10 with 20 units/mL of IL-2 every 3 or 4 days of incubation. HIV-1 replication activity was determined by measuring p24 antigen levels in the culture supernatants using the p24 antigen capture assay (PerkinElmer, Boston, MA, USA) [24].

### 2.6. Western Blotting

To confirm the proteolytic processing of Gag and GagPol polyproteins in HIV virions, Western blot (WB) analysis was performed as previously described [22,24]. Due to the abnormal sizes of M50I mutant particles (the diameters of the mutant particles were 190~300 nm, while those of Wt were 110~130 nm [22]) with a defective autoprocessing of Gag and GagPol polyproteins (lacking of the cleaved mature forms of MA (p17), CA (p24), PR, RT, and IN in the virus particles) [22], we could not find an appropriate internal control for WB. Thus, we used 1 µg of total viral protein to demonstrate impaired autoprocessing as previously described [22,24]. WB was performed using a rabbit polyclonal anti-Anti-HIV1 p17/p24/Gag antibody (Cat# ab63917, Abcam, Waltham, MA, USA), a mouse monoclonal anti-HIV p24 antibody (Cat# ab9071, Abcam), and a rabbit polyclonal anti-PR antibody (Cat# ab211627 Abcam, Cat# SKU:65-018, As One International, Santa Clara, CA, USA). Protein bands were detected by using the ECL Prime Western Blotting Detection Reagent (MiliporeSigma) with the Azure 300 (Azure Biosystem (Dublin, CA, USA) [22].

### 2.7. Structure Analysis

The HIV Cryo-EM structure of the PR-RT structure (PDB accession #: 7sjx) [35] was downloaded from the PDB database (https://www.rcsb.org, accessed on 29 August 2022) into PyMOL (https://pymol.org/, version 2.5.4, accessed on 29 August 2022). Chain A in the structure was colored in lime (the RH part was colored in cyan), and chain B was colored in olive. RH:N79N and RH:D109 were only visible in chain A (Chain A:D630), colored in red and blue, respectively. The distance of the alpha carbon between RH:N79 and RH:D109 was calculated using the following command: distance chain A and i. 600 and n. CA, chain A and i. 630 and n. CA.

### 2.8. Statistical Analysis

Intergroup comparisons were performed by one-way analysis of variance (ANOVA) with multiple comparison analysis or Student’s *t*-test using GraphPad Prism 9 (GraphPad, San Diego, CA, USA). *p*-values less than 0.05 were considered statistically significant (* *p* < 0.05, ** *p* < 0.01, *** *p* < 0.001, **** *p* < 0.0001, *p* > 0.05 was considered not significant (ns).

## 3. Results

### 3.1. The C-Terminal Domain of RNase H in the GagPol Polyprotein Regulates the Virus Release and Autoprocessing Defects in HIV(IN:M50I/V151I)

**Figure 1:**
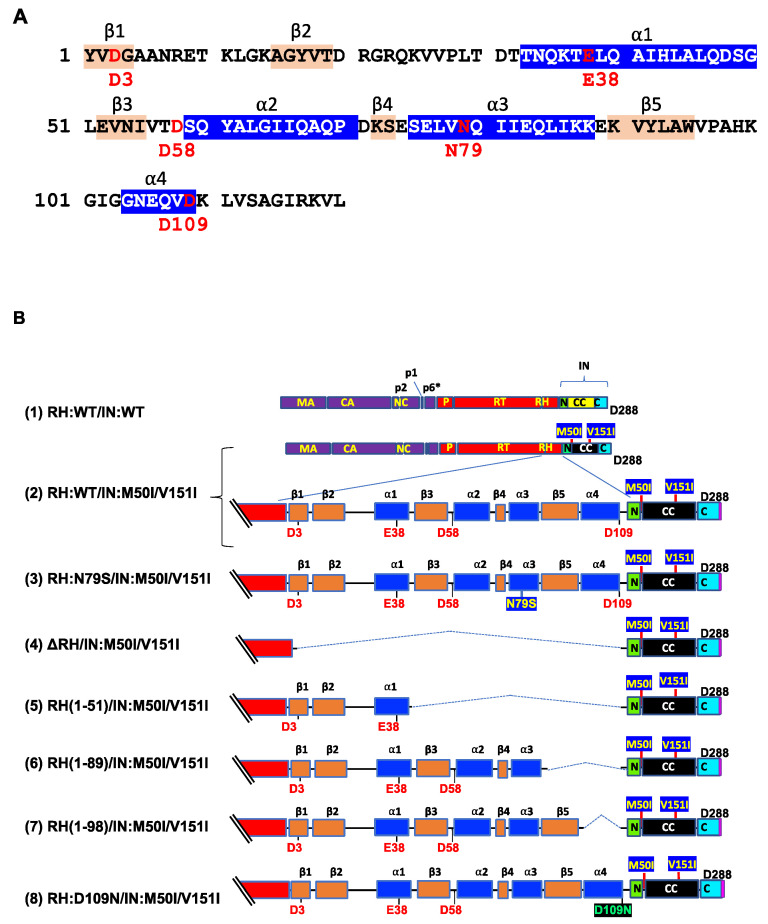

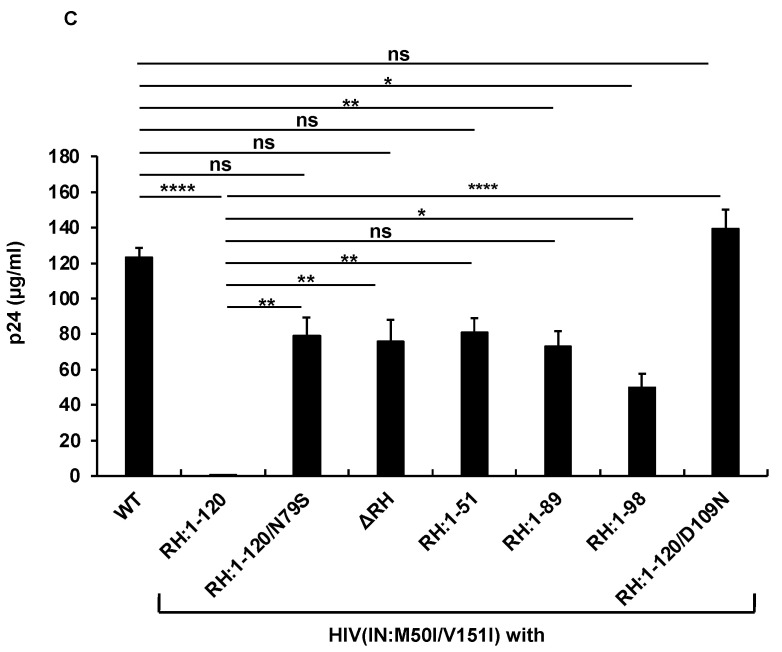
The C-terminal domain of RH regulates virus release and autoprocessing. (**A**) Amino acid sequence of RH. Amino acid sequence of RH of HIV_NL4.3_ [33] was obtained from GenBank (accession #: AF324493). The alpha helices are colored in blue, and the beta strands are colored in wheat; codons associated with the active sites (D3, E38, D58, and D109) and mutation position 79 (N79) are labeled and colored in red with numbering. (**B**) Diagrams of HIV mutants containing mutations in RH. The diagram indicates the structure of GagPol(WT) and variants. The numbers above the diagrams indicate codon positions in RH. Each mutant was created by inverting PCR or site-directed mutagenesis, as described in the Materials and Methods (Section 2.3). MA: matrix protein; CA: capsid protein; NC: nucleocapsid protein; P: protease; RT: reverse transcriptase; RH: RNase H; IN: integrase; N: the N terminal domain of IN; CC: the catalytic core domain of IN; C: the C terminal domain of IN; α1~α4 and β1~β5 are corresponding to domains of RH in Figure 1A. (**C**) Each construct was transfected into HEK293T cells. Transfection supernatants from HEK293T cells were collected, and then viral particles were pelted as described in the Materials and Methods (Section 2.4). Each viral pellet was resuspended in 1/100 of the transfection supernatants in complete culture media or PBS. p24 concentrations in the suspension were determined by measuring HIV p24 antigen by ELISA. Data indicate means ± SE (n = 5), and statistical analysis was conducted using one-way ANOVA (Prism). *: *p* < 0.05, **: *p* < 0.01, ****: *p* < 0.0001, ns.: not significant.

We reported that RH:N79S or IN:S17N restored the inhibition of virus release and autoprocessing of HIV(IN:M50I/V151I) [22]. In the current study, we attempted to determine whether the domains containing the changes directly regulated the virus release and initiation of the autoprocessing. RH consists of four α-helixes (α1~α4 helixes) and five β-sheets (β1~ β5 sheets), and the codon 79 is located in the middle of the α3 helix (Figure 1A, Supplemental Figure S1) and forms the active pocket with a highly conserved DEDD motif (the motif is composed of D3 on the β1-sheet, E38 on the α1-helix, D58 on the loop between the β3-sheet and the α2-helix, and D109 on the α4-helix), which coordinates two divalent cations (Mn^2+^ or Mg^2+^) [36] required for hydrolyzing the HIV genomic RNA substrate. Although the compensatory mutation, RH:N79S, is located on the α3 helix, which is outside of the metal binding pocket with 15.3~28.3Å distance from the activation site (Supplemental Figures S1 and S2), it rescued virus release and initiated autoprocessing followed by virus replication [22].

**Figure 2:**
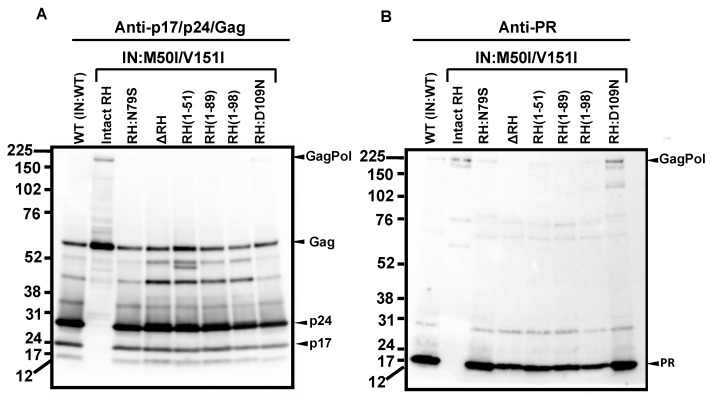

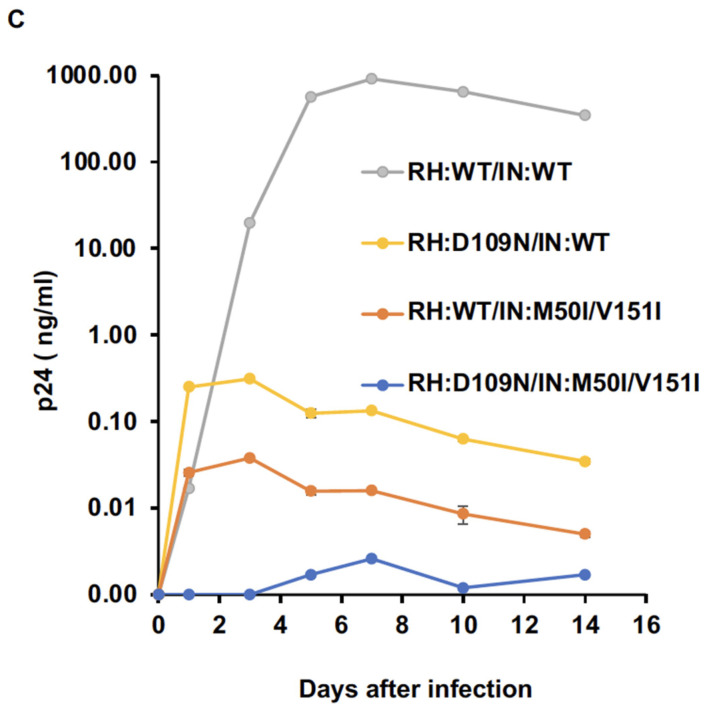
RH:D109 regulates virus release and autoprocessing in the presence of IN:M50I/V151I. (**A**,**B**) Virus particles are isolated using ultracentrifugation, as described in the Materials and Methods (Section 2.4), and 1 μg of viral lysates was subjected to WB. Gag/GagPol-cleaved products and PR were detected by rabbit polyclonal anti-p17/p24/Gag (**A**) and anti-PR (**B**), as described in the Materials and Methods section. (**C**) PHA-stimulated primary CD4(+) T cells obtained from healthy donors were infected with 10 ng p24 amounts of HIV(WT) or variants, as described in the Materials and Methods (Section 2.6). The infected cells were cultured for 14 days with a changing medium every 3 to 4 days. HIV replication was monitored using a p24 antigen capture kit. Representative data from three independent assays are presented as means ± standard deviations (SDs) (n = 3).

Thus, RH:N79S may trigger a structural change in IN:M50I/V151I GagPol; subsequently, it may induce the restoration of autoprocessing followed by maturation. Given this notice, we speculated that a particular domain(s)/region(s) of RH may be directly associated with the recovery. We decided to determine how the RH domain(s) in the M50I/V151I mutant influenced autoprocessing and the virus maturation and constructed a series of HIV(M50I/V151I) variants containing different lengths of RH (Figure 1B). To evaluate the processing function of PR, we maintained the PR cleavage sequence at the junction between RH and IN in all variants; all mutants retained five amino acid (aa) upstream sequences of the PR cleavage site from the C terminal of RH. Each plasmid construct was transfected into HEK293T cells. The released virus particles in the culture supernatants were collected by centrifugation and then resuspended in PBS or fresh culture medium, as described in the Materials and Methods (Section 2.4). The amounts of released virus particles were quantified using a p24 antigen capture assay. The processing of Gag and GagPol in the particles was analyzed by Western blot (WB) assays using anti-Gag or anti-PR antibodies. Consistent with our previous results, the amounts of HIV(IN:M50I/V151I) released were suppressed to 0.23 + 0.02% (n = 5) (*p* < 0.0001) compared to HIV WT (Figure 1C) and restored to 64 + 0.1% (n = 4) (*p* > 0.05) of HIV(WT) in the presence of RH:N79S. The GagPol processing was also rescued (Figure 2A,B). All variants lacking the α4 helix domain could recover the virus release to 40~60% of WT (Figure 1C, Supplemental Table S2). The processing activity was recovered, and the mature form of PR was detected in all mutants, even in the absence of the RH:N79S (Figure 2A,B). The result indicated that the α4- rather than the α3-helix domain played a key role in regulating the virus release from the HIV-1-producing cells and the autoprocessing occurring in HIV(IN:M5I/V151), and it negatively regulates both processes.

The α4 helix domain comprises of six aa sequences (Gly–Asn–Glu–Gln–Val–Asp, Figure 1A) containing RH:D109. The residue forms a metal-binding pocket in RH with three other residues (D3, E38, D58) [36,37]. To define the key amino acid residue(s) present in the domain, we performed a population analysis using HIV sequence data from the Los Alamos National Laboratory (LANL, http://www.hiv.lanl.gov, accessed on 13 July 2022), Stanford drug-resistance database (https://hivdb.stanford.edu/, accessed on 17 November 2021), and NIAID HIV sequence data. The population analysis indicated that RH:D109 was highly conserved among the databases (LANL database: 99.62%, Stanford database: 100%, NIAID: 100%, Supplemental Table S3) [36]; thus, we became interested in defining how RH:D109 affects the defective virus. The mutation Asp-to-Asn change at codon 109 (RH:D109N) was produced in HIV(IN:M50I/V151I) by site-directed mutagenesis, and then the quantities of the released virus and Gag/GagPol processing were analyzed. Interestingly, the mutation comparably restored the virus release (Figure 1C, Supplemental Table S2) and the autoprocessing with HIV(WT) (Figure 1D,E); however, virus replication was defective (Figure 2C, Supplemental Figure S3) due to the lack of a functional metal-binding pocket.

**Figure 3:**
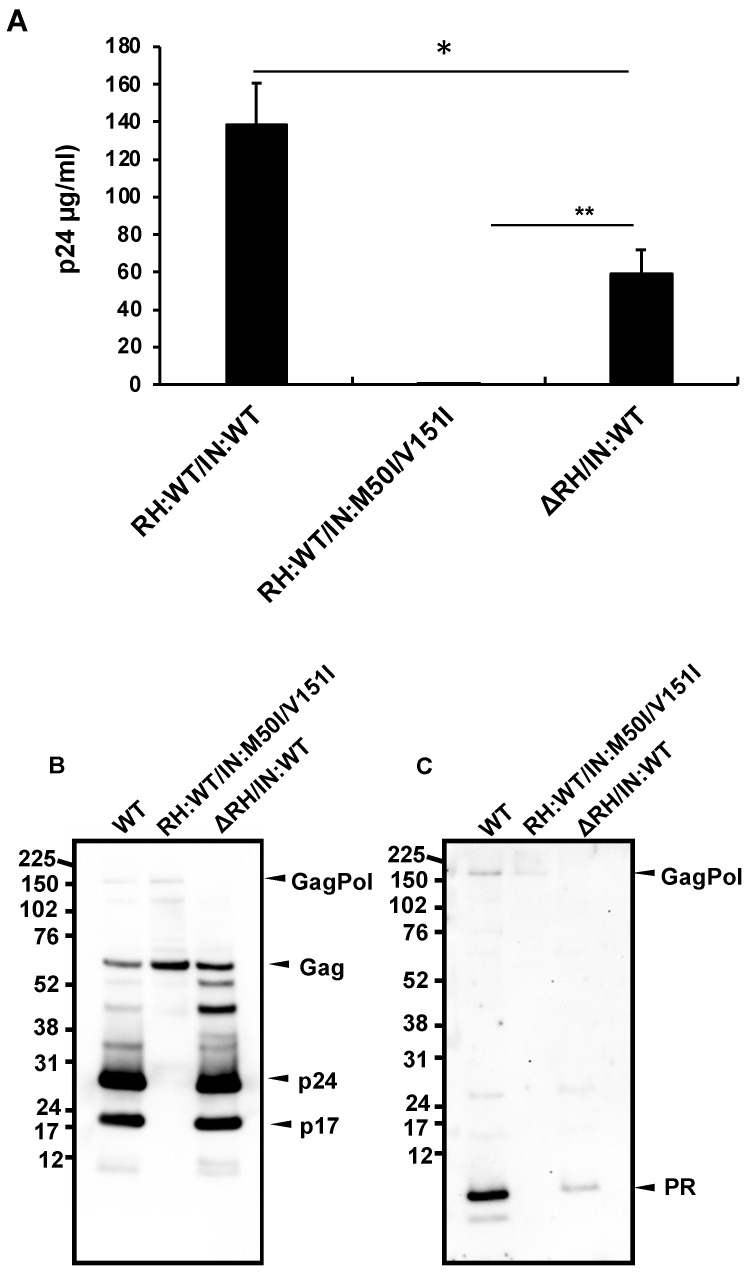
The C-terminal domain of RH regulates virus release and autoprocessing in HIV(IN:WT). (**A**–**C**) Each construct was transfected into HEK293T cells. Transfection supernatants were collected, and viral particles in the supernatants were pelleted as described in the Materials and Methods (Section 2.4). Each viral pellet was resuspended in 1/100 of the transfection supernatants in complete culture media or PBS. (**A**) P24 concentration in the suspension was determined by measuring HIV p24 antigen by ELISA. Data indicate means ± SEs (n = 5); statistical analysis was conducted using one-way ANOVA (Prism). (**B**,**C**) Virus particles are isolated as described in the Materials and Methods (Section 2.4). 1 μg of viral lysates was subjected to WB. Gag/GagPol-cleaved products and PR were detected by rabbit polyclonal anti-p17/p24/Gag (**B**) and anti-PR antibodies (**C**). *: *p* < 0.05, **: *p* < 0.01.

The defect of HIV(IN:M50I/V151I) in the virus-release process was partially rescued in the absence of RH (Figure 1C), implicating that the RH domain in the GagPol negatively regulates the virus release in the defective virus. To define whether the RH domain affects the virus release of HIV(IN:WT), the RH domain-deleted HIV(IN:WT), HIV(ΔRH/IN:WT) was created, and the virus release was assessed. If the RH domain negatively regulated the virus release, we expected that the release of HIV(ΔRH/IN:WT) might be increased; however, the mutant demonstrated 53 ± 11% (n = 3, *p* < 0.05) of HIV(WT) (Figure 3A), indicating that the RH domain positively regulates the virus release of HIV(WT). WB demonstrated that HIV(ΔRH/IN:WT) was able to process Gag and GagPol (Figure 3B,C); thus, the RH domain is involved in virus release.

### 3.2. Asp 288 at the C-Terminal of Integrase (IN) Is a Key Player in the M50I/V151I Defects in Autoprocessing and Virus Release

**Figure 4:**
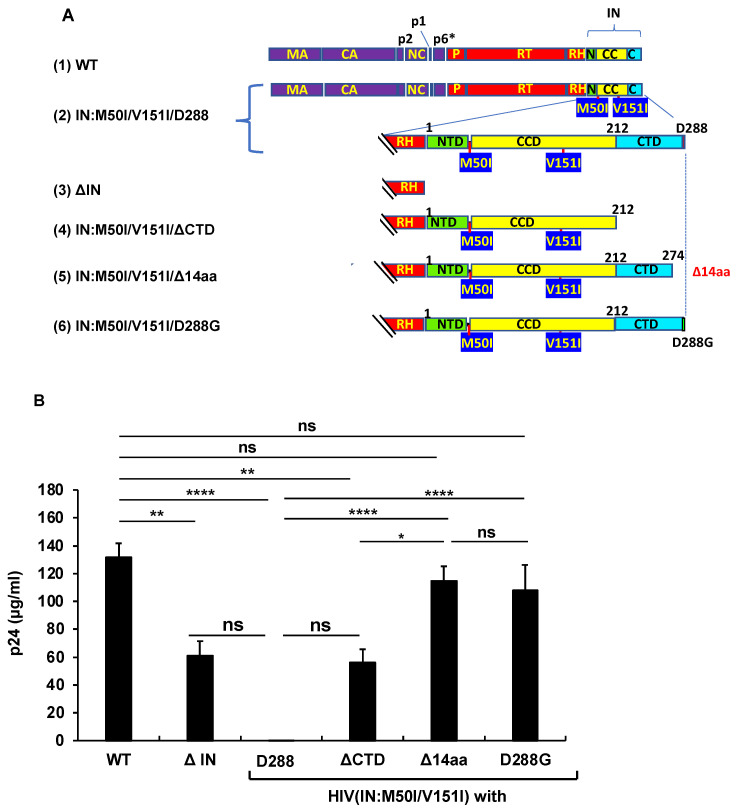

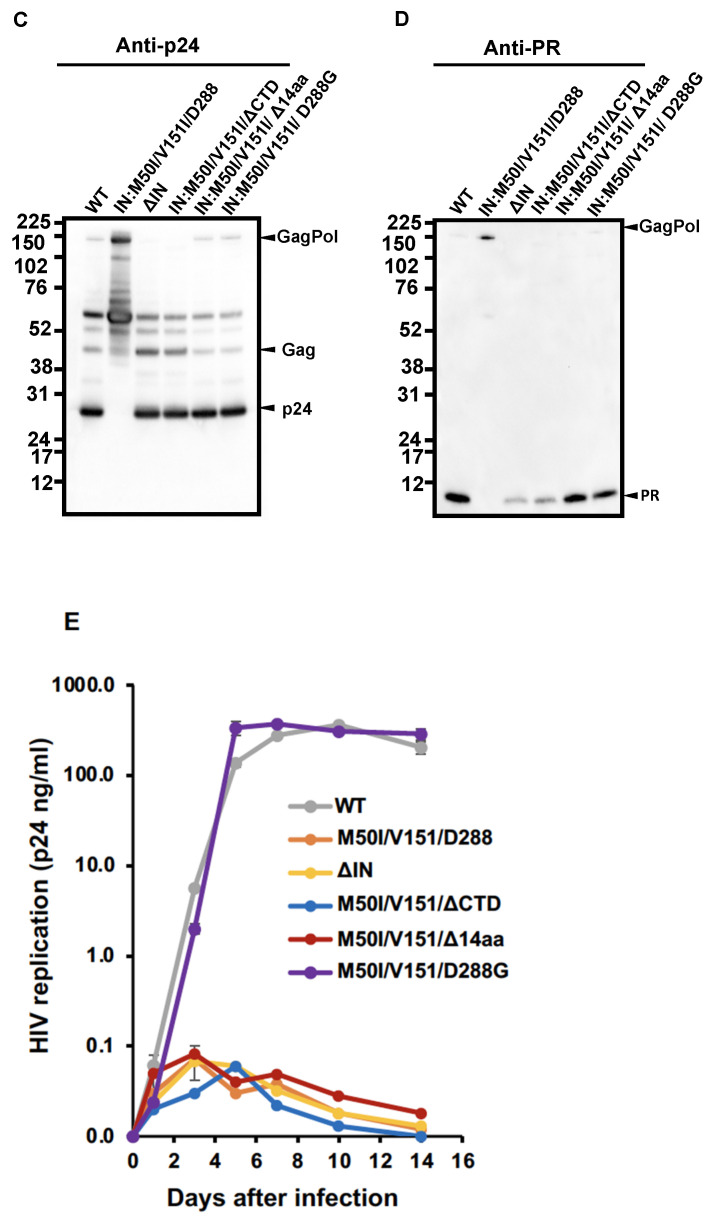
The C-terminal domain of IN regulates virus release and autoprocessing. (**A**) Diagrams indicate the structure of GagPol(WT) and variants. The numbers above the diagrams indicate codon position in IN. Each mutant was created by inverse PCR or site-directed mutagenesis, as described in the Materials and Methods (Section 2.3). MA: matrix protein; CA: capsid protein; NC: nucleocapsid protein; P: protease; RT: reverse transcriptase; RH: RNase H; IN: integrase; N: the N terminal domain of IN; CC: the catalytic core domain of IN; C: the C terminal domain of IN. (**B**) Each construct was transfected into HEK293T cells. Transfection supernatants derived from HEK293T cells were collected, and viral particles were pelleted as described in the Materials and Methods (Section 2.4) Each viral pellet was resuspended in 1/100 of the transfection supernatants in complete culture media. Viral concentration was determined by measuring the HIV p24 antigen by ELISA. Data indicate means ± SE (n = 5), and the statistical analysis was conducted using one-way ANOVA (Prism). (**C**,**D**) Virus particles are isolated as described in Materials and Methods (Section 2.4), and 1 μg of viral lysates was subjected to WB. Gag and GagPol-cleaved products and PR were detected by monoclonal anti-p24 (**C**) and polyclonal anti-PR (**D**). (**E**) PHA-stimulated primary CD4(+)T cells from healthy donors were infected with 10 ng p24 amounts of HIV(WT) or variants containing mutations as described in Materials and Methods (Section 2.5). The infected cells were cultured for 14 days with a medium change every 3 to 4 days. HIV replication was monitored using a p24 antigen capture kit. Representative data from two independent assays are presented as means ± standard deviations (SD) (n = 3). *: *p* < 0.05, **: *p* < 0.01, ****: *p* < 0.0001, ns: not significant.

As shown above, RH:D109N rescued the defects in autoprocessing and virus release in the context of HIV(IN:M50I/V151I). This implicated that the residue or the α4 helix domain of RH negatively regulated both the virus release and the initiation of autoprocessing in the context. To further understand the suppression mechanism in the presence of RH:D109 and the role of domains of IN in inducing the defect, we constructed a series of variants (Figure 4A). IN is composed of three domains, the N-terminal domain (NTD), the catalytic core domain (CCD), and the C-terminal domain (CTD) [38,39,40]. Codon 50 is located at the linker/hinge region between NTD and CCD, which is 21~25 angstrom away from the activation site of IN (Supplemental Figure S4). In contrast, codon 151 exists in CCD and is a neighbor of acidic residues in the active pocket. Thus, we focused on CTD to construct the mutants to maintain the M50I/V151I effect. Then, virus release and autoprocessing were analyzed.

The virus release of the mutant lacking the entire IN (ΔIN) was suppressed to 49.7 ± 8.5% of that of WT (*p* < 0.05, n = 4) (Figure 4B), and the WB results demonstrated that it partially suppressed autoprocessing (Figure 4C,D). Therefore, consistent with the results of the previous reports [7,9], IN was proved to be involved in regulating autoprocessing. A mutant lacking the entire CTD (HIV(M50I/V151I/ΔCTD) partially restored the virus replication (45.5 ± 7.9% of WT, *p* < 0.05, n = 4) and autoprocessing (Figure 4C,D). Of interest, a mutant without the C-terminal tail of 14 aa sequences of IN, HIV(IN:M50I/V151/Δ14aa), restored the virus release and processing to comparable levels of WT (Figure 4B–D), in comparison to WT, indicating that the last 14 aa sequences (Met-Ala-Gly-Asp-Asp-Cys-Val-Ala-Gly-Arg-Gln-Asp-Glu-Asp) regulate both autoprocessing and the virus release of HIV(IN:M50I/V151I) in the presence of RH:D109.

We also assessed the capability of HIV replication and compared its activity among the mutants. It is reported that variants lacking the last 15~17 aa residues at the tail domain diminished enzymatic function in IN and infectivity [20,41]; this was consistent with the reports that all mutants lacking IN domains did not indicate a detectable level of HIV replication (Figure 4E).

We considered how the tail restored the autoprocessing, even in the presence of RH:D109. Crystal structural analysis of the full length of IN has not yet been successful because of the flexibility and instability of the tail, and its poor solubility under low salt conditions [35,38,42,43,44]. It was assumed that the tail of IN:M50I/V151I might directly (e.g., structure hindrance) or indirectly (e.g., interaction with host proteins) influence the initiation of the autoprocessing at the junction of p2 and NC. If an unknown factor was involved in the defect, we thought that the last amino acid residue might critically function, rather than the middle of the amino acid residues in the tail. Asp is an acidic amino acid; thus, we presumed the charge or size of the residue might influence the defect. To address the hypothesis, we chose an aa change at 288 from Asp to Gly, the smallest aa side residue without polar, as a pilot experiment. Using site mutagenesis, we constructed a mutant, HIV(IN:M50I/V51I/D288G), and then, virus release, autoprocessing, and virus replication assays were carried out. Surprisingly, the variant demonstrated indistinguishable activities in the virus release (Figure 4B, Supplemental Table S4), autoprocessing (Figure 4C,D), and virus replication (Figure 4E), compared to WT. These data indicated that the last amino acid of the tail of IN regulates viral infectability in the IN:M50I/V151I mutant setting with RH:D109.

**Figure 5:**
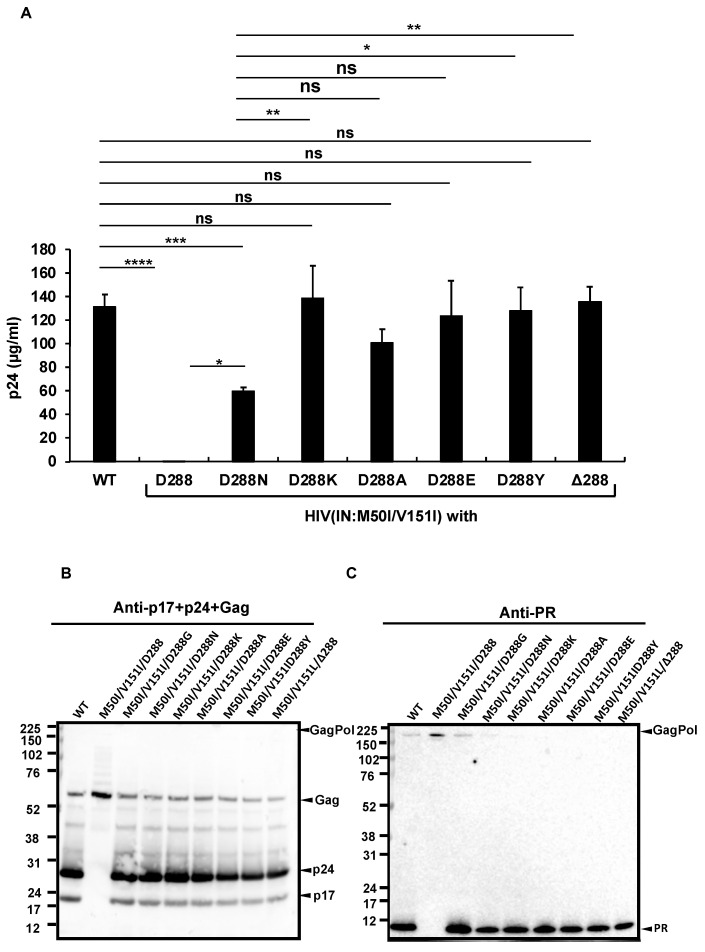

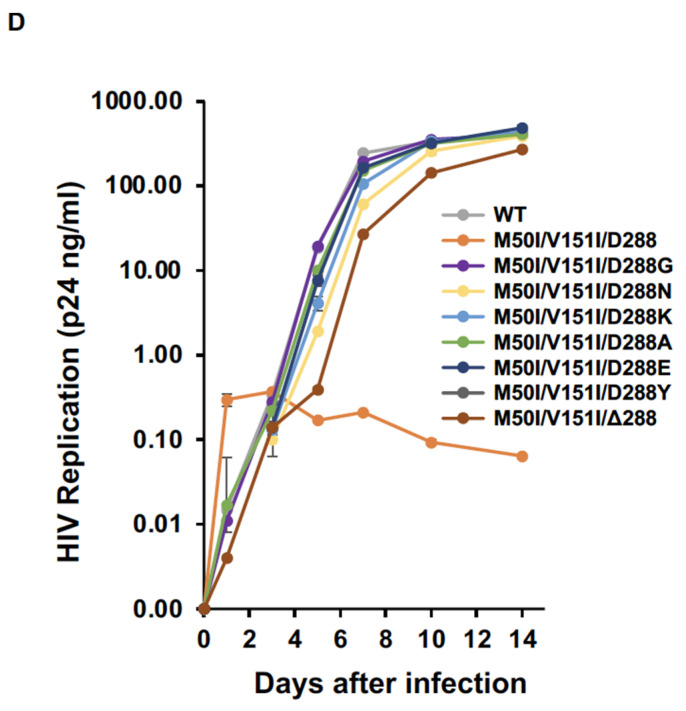
The C-terminus amino acid residue, Asp, regulates virus release and processing of HIV(M50I/V151I). (**A**) Each construct was transfected into HEK293T cells. Transfection supernatants from the HEK293T cells were collected, and viral particles were pelted as described in the Materials and Methods (Section 2.4). Each viral pellet was resuspended in 1/100 of the transfection supernatants in complete culture media. Viral concentration was determined by measuring HIV p24 antigen by ELISA. Data indicate means ± SEs (n = 5). Statistical analysis was conducted using one-way ANOVA. (**B**,**C**) Each virus particle was isolated from the transfection supernatant as described in the Materials and Methods (Section 2.4), and 1 μg of each viral lysate was subjected to WB. Gag- and GagPol-cleaved products and PR were detected by polyclonal anti-p17/p24/gag (**B**) and anti-PR (**C**). (**D**) PHA-stimulated primary CD4(+) T cells derived from healthy donors were infected with 10 ng p24 amounts of HIV(WT) or variants containing mutations, as described in the Materials and Methods (Section 2.5). The infected cells were cultured for 14 days with the medium change every 3 to 4 days. HIV replication was monitored using a p24 antigen capture kit. Representative data from three independent assays are presented as means ± SDs (n = 3). *: *p* < 0.05, **: *p* < 0.01, ***: *p* < 0.001, ****: *p* < 0.0001, ns.: not significant.

The D288G change alters the net charge and size of the aa side-residue at the tail. The LANL, Stanford, and NIAID HIV sequence databases demonstrated that Asp at 288 in IN (IN:D288) is relatively conserved in HIV sequences (the percentages of populations of Asp at 288 in LANL, Stanford, and NIAID databases were 93.7%, 97.85%, and 96.12%, respectively) (Supplemental Table S5); thus, to further define a correlation between the side residue and virus release/autoprocessing/virus replication, we produced a series of HIV(IN:M50I/V151I) mutants containing amino acid changes at codon 288. Neutral, basic, and hydrophobic amino acid changes were chosen from mutations detected in the clinical isolates (Supplemental Table S5). In addition, to address the role of a polar side chain at 288, D288Y was also introduced by site-directed mutagenesis. The virus release of mutants containing D288K, D228A, D228E, or D288Y was restored to a comparable level of HIV(WT). A mutant containing D288N restored the virus release to only 50% of HIV(WT) (*p* < 0.05) (Figure 5A); however, the processing levels in all mutants were similar to that of WT (Figure 5B,C), and virus replication was rescued in all mutants (Figure 5D), indicating that the last amino acid Asp at 288 may play a key role in regulating virus-release activity.

To confirm the function of the last codon, we created another mutant lacking the codon in the setting of context, HIV(IN:M50I/V151/Δ288). Intriguingly, the virus lacking the last amino acid of IN could fully recover virus release and autoprocessing activities (Figure 5A). However, the replication capability of the mutant on day 7 after infection was 10~20% of that of WT (Figure 5D), indicating that the last aa residue may not be required for regulating virus release and autoprocessing but is the essential residue for virus replication in the setting of the IN:M50I/V151I mutant.

### 3.3. Population Analysis of the C-Terminal Amino Acid of the Combination of IN:M50I/V151I in the HIV Sequence Database

**Figure 6:**
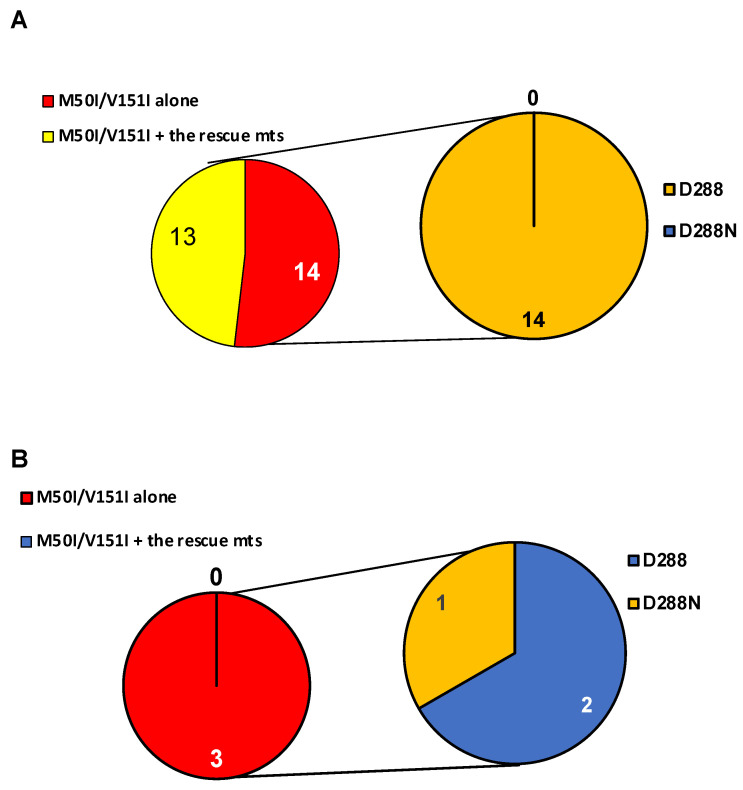
Population analysis of a combination of IN:M50I/IN:V151I mutations with an aa residue at 288 of IN. Population analysis was conducted to determine the diversity of HIV sequences containing IN:M50I and IN:V151 in (**A**) subtypes B, and (**B**) subtype C in the LANL HIV database. All HIV-1 sequences were downloaded from the LANL.GOV website. The sequences containing IN (p31) regions were selected, and only one sequence per patient was used in the analysis. A total of 27 and 3 HIV sequences contained IN:M50I/V151I mutations in subtype B (**A**) and C (**B**), respectively. The aa residue at 288 was analyzed in sequences without rescue mutations (RH:N79S or IN:S17N).

As described in the Introduction section, we had previously reported that the RH:N79S or IN:S17N mutation rescued the defect of HIV(IN:M50I/V151I) replication [1,2]. In the study, we conducted a population analysis in the sequenced samples of NIAID and the Los LANL database to define the population of IN:M50I/V151I mutants containing the rescue mutations. We reported that the M50I/V151I mutation was only detected in the LANL database (27 out of 6335 subtype-B and 3 out of 2871 subtype-C sequences) but not in the NIAID samples, and interestingly, 14 of 27 subtype-B and all 3 subtype-C sequences did not possess the rescue mutations [24]. We performed an additional population analysis in the current study to define aa residue at 288 in the 14 subtype-B and the 3 subtype-C sequences. Only 1 sequence in subtype C possessed IN:D288N, but the other 16 sequences contained D288D (Figure 6A,B).

## 4. Discussion

We have previously demonstrated that a combination of M50I and V151I mutations in IN suppresses virus release by accumulating the abnormal size of buds on the surface of HIV-producing cells. In the presence of the combination, the processing of Gag and GagPol was inhibited at the initiation of autoprocessing [22,24]. RH:N79S or IN:S17N rescued the defect; however, the mechanism of the defective virus is not yet clear. Given the information, in the current study, using truncated mutants, we further investigated the role of each RH and IN domain in the impaired virus. We found that RH:D109 and IN:D288 negatively regulate the defects occurring in the M50I/V151I setting. RH:D109 is a component to form the active pocket of RH and is highly conserved [36]. The sequential deletion of RH in HIV(IN:M50I/V151I) demonstrated that the α4 domain containing RH:D109 regulates the defect of HIV(IN:M50I/V151I). The RH:D109N change in HIV(IN:M50I/V151I) rescued the processing of Gag and GagPol, but significantly suppressed virus fitness. Therefore, a combination of RH:D109 with IN:M50I/V151I induces a defect in the initiation of autoprocessing. Although we illustrated that RH:D109N in HIV(IN:WT) was replication-incompetent, to precisely elucidate the role of the domain in HIV replication, we need further studies using HIV(M50I/V151I) and HIV(IN:WT).

A series of HIV(IN:M50I/V15I) variants containing aa substitutions at IN:D288 illustrated that six aa changes (Gly, Asn, Lys, Ala, Glu, Tyr) could play a role as compensatory mutations for virus release, autoprocessing, and virus fitness of HIV(IN:M50I/V151I) without the previously described rescue mutations. In order to define the clinical settings, we conducted a population analysis using NIAID HIV sequences and the LANL HIV database. We previously reported that 17 sequences in LANL (14 subtype B and 3 subtype C) contained IN:M50I/V151I mutations without the rescue mutations [24]. We expected that the 17 sequences contained aa changes at D288; however, only one out of 17 contained D288N mutation, and the others had no change in the sequence. It appears that the 16 sequences may carry other uncharacterized compensatory mutation(s).

The results of WB from HIV(ΔIN) and HIV(IN:M50I/V151/ΔCTD) demonstrated that both mutants contained less PR than HIV(WT). By contrast, HIV(IN:M50I/V151I/Δ14aa) and HIV(IN:M50I/V151I/D288G) carried a comparable amount of PR to HIV(WT) (Figure 4). The band intensity of p24 in the WB results from all mutants was similar to that of HIV(WT). Therefore, the loaded protein amounts of the mutants in the WB assay were similar, indicating that HIV(ΔIN) and HIV(IN:M50I/V151/ΔCTD) particles might contain less incorporated GagPol. Both mutants commonly lack aa residues 212–274; thus, the region may be associated with GagPol incorporation in the particles. The IN-deletion studies report that mutations in CCD (aa residues 58–202) regulate GagPol incorporation [19,43,45,46]. Uncharacterized aa residues in the 212–274 region may regulate GagPol incorporation in the virus particles with a similar mechanism by the mutations in the CCD.

The sequential deletion of aa residues from the C-terminal of IN(WT) revealed that the IN C-terminal tail region (aa residues 270–288) is required for HIV-1 infection, reverse transcription, and maturation [17,20]. A functional study of IN using recombinant protein identified that Lys273 in the tail region takes part in virus RNA binding with other residues [47]. Our findings provide a further insight into the regulation of virus release and maturation by amino-acid-substituted IN and the tail region of HIV(IN:M50I/V151I). Inhibitors of HIV maturation have been considered as the next-generation anti-virus drugs [48,49]; thus, our finding may provide new insight into developing new drugs.

Autoprocessing is initiated at the junction between p2 and NC in intramolecular mechanisms [12]. In our previous work, we demonstrated that HIV(IN:M50I/V151I) suppressed this step without affecting GagPol dimerization [22], indicating that GagPol-containing IN:M50I/V151I may directly or indirectly suppress the autoprocessing after the dimerization. Due to its flexibility and poor solubility of the C-terminal domain of IN, the structure analyses were performed using the tail region-deleted IN, not the intact, full-length of IN [35,38,42,43,44]. To understand the mechanism of the defect of the initiation of autoprocessing in HIV(IN:M50I/V151I), we need to structure an analysis of the full-length of GagPol. Our previous study illustrated that HIV(IN:M50I/V151I), with a modification at the junction between PR and RT in the GagPol, restored the processing [22], suggesting that the tail domain of IN: M50I/V151I may directly or indirectly interact with the junction and induce a conformation change in the GagPol structure. Subsequently, it may inhibit the initiation of autoprocessing. HIV-1 particles incorporate a large array of host proteins during assembly and budding [50,51,52,53,54,55], and those proteins may positively or negatively regulate virus life cycles. The M50I/V151I particles may incorporate a unique host protein via the C-terminal domain of IN:M50I/V151I and interfere with the initiation of autoprocessing, or may lack the incorporation of the host proteins requiring budding [55] via the IN, subsequently suppressing virus release. Currently, a comparative proteomic analysis of HIV particles between HIV(WT) and HIV(IN:M50I/V151I) is underway, which may reveal the regulatory mechanism(s) of this defect.

Using RH-truncated mutants in HIV(IN:WT) and HIV(IN:M50I/V151I), our study demonstrated that the RH domain is involved in virus release; ΔRH suppressed HIV(IN:WT) release, but ΔRH partially rescued HIV(IN:M50I/V151I) release. In contrast, the WB results demonstrated that the amounts of Gag/GagPol processing in both ΔRH mutants were comparable to that of HIV(WT). It appears that the RH domain is not involved in regulating the initiation of autoprocessing. IN:D288G/K/E/Y fully restored virus release, autoprocessing, and viral replication. The IN:D288N change also nearly fully recovered the processing, however, it partially rescued the virus release of HIV(IN:M50I/V151I) to 48.6 ± 6.3% of that of HIV(WT) (*p* < 0.05, n = 5). In addition, a mutant lacking codon 288, HIV(IN:M50I/V151I/Δ288), was able to restore virus release and autoprocessing, but virus replication was 10~20% of HIV(WT) on day 7. Therefore, the virus release, autoprocessing, and virus replication may be independently regulated in a C-terminal aa-dependent manner.

HIV particles are budding and released from the cell surface by recruiting the host ESCRT system [9,56,57,58,59]. Near 20 ESCRT proteins and other host factors are involved in the process [9,10,11,60,61,62]. Thus, the GagPol-containing IN:M50I/V151I may differentially regulate the ESCRT system and suppress virus release. Recent studies highlight the accessory functions of matured IN [47,63,64]: IN stimulates RT [63], IN interacts with RNA [47,65,66], the acetylation of CTD of IN is associated with the defects in proviral transcription [65]. We now demonstrated a new potential role of IN domain in GagPol polyprotein.

We previously observed that HIV(IN:M50I/V151I) was defective in virus release and autoprocessing followed by maturation; thus, the mutant was replication-incompetent [22,24]. The current study focused on determining the amino acid residue(s) that regulate the defects. We found that the C-terminal domains of RH and IN play a pivotal role in the impaired virus. Further studies are required to define the role of each aa in virus replication. However, site-directed mutagenesis study in the M50I/V151I context demonstrated that some mutations partially restored virus release and rescued a nearly complete level of the processing. These results imply that virus release is tightly correlated with autoprocessing (the initiation of processing), and autoprocessing may trigger virus release. Recently, Tabler et al. demonstrated that the embedded PR in GagPol is activated during assembly and budding prior to virus release [16], indicating that PR activation is related with the virus release process. Our results are in line with their report. Further investigations need to reveal the regulatory mechanism of virus release and budding, focusing on the C-terminal domains of RH and IN, which may provide a feasible strategy for the suppression of virus transmission, and may provide a novel insight into developing therapy.

## Data Availability

Not applicable.

## Supplementary Materials

The following supporting information can be downloaded at: https://www.mdpi.com/article/10.3390/v14122687/s1, Figure S1: Structure of RNase H in PR-RT fusion protein; Figure S2: Calculation of a distance between RH:N79 and RH:D109; Figure S3: Impact of RH:D109N change on HIV replication in PHA-stimulated primary CD4(+) T cells; Figure S4: 3D structure of IN (WT); Table S1: DNA sequence of primers for mutagenesis; Table S2: The *p* values of combination of RH variants with IN:M50I/V151I/D288; Table S3: Population analysis of Codon 109 of RH in Database; Table S4: The *p* values of combination of IN variants with IN:M50I/V151I; Table S5: Percentage of each amino acid residue in total analyzed sequences.
