# Supplementary material for: The C-Terminal Domain of RNase H and the C-Terminus Amino Acid Residue Regulate Virus Release and Autoprocessing of a Defective HIV-1 Possessing M50I and V151I Changes in Integrase
Source: Viruses. 2022 Nov 30;14(12):2687. doi: 10.3390/v14122687 (PMC9788298; doi:10.3390/v14122687)
Supplement: Supplementary file 1 [file viruses-14-02687-s001.zip › Supplemental Figures S1-S4.pdf]

Supplemental Figure S1

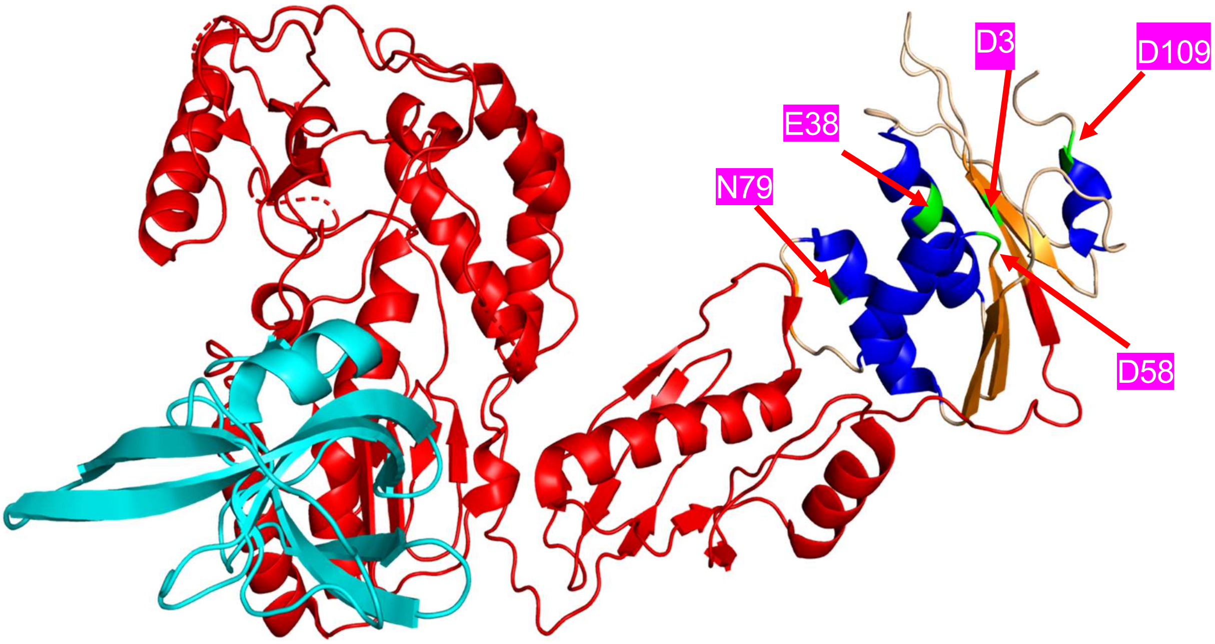

Figure S1. Structure of RNase H in PR-RT fusion protein
The HIV POL structure 7SJX were downloaded directly from the PDB database (https://www.rcsb.org/)into PyMOL and chain B was removed. The remaining chain A was colored based on its components: PR as cyan and RT as red, and the RH part of chain A was colored based on the secondary structure (Helix: Blue; beta sheet: orange and loop: wheat). The key amino acids were colored as green and labeled.

Supplemental Figure S2

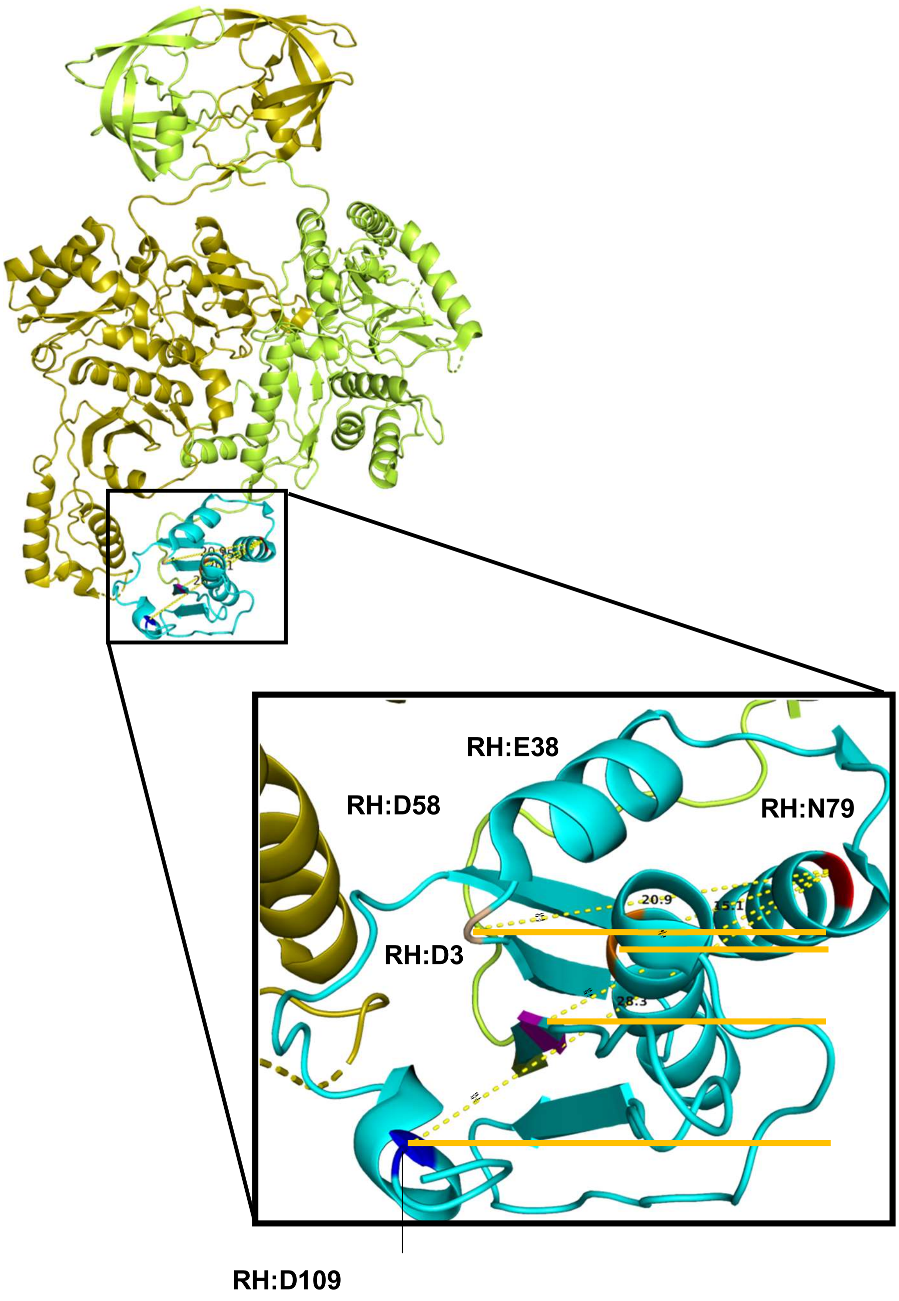

Figure S2. Calculation of a distance between RH:N79 and RH:D109
The HIV Cryo-EM Structure of the PR-RT structure (PD accession #: 7SJX) was downloaded from the PDB database (https://www.rcsb.org) into PyMOL (https://pymol.org/2). Chain A in the structure was colored with limon (RH is shown with cyan) and chain B was colored with olive (RH is not visible in Chain B). The RH:N79 (Chain A:D600), RH:D3 (Chain A:D524) , RH:E38 (Chain A:D559), RH:D58 (Chain A:D579), and RH:D109 (Chain A:D630) colored as red, magenta, orange, wheat and blue. Distance of alpha carbon between RH:N79 and each residue were calculated with the command: distance chain A and i. 600 and n. CA, chain A and i. (position B coordinate) and n. CA.

Supplemental Figure S3.

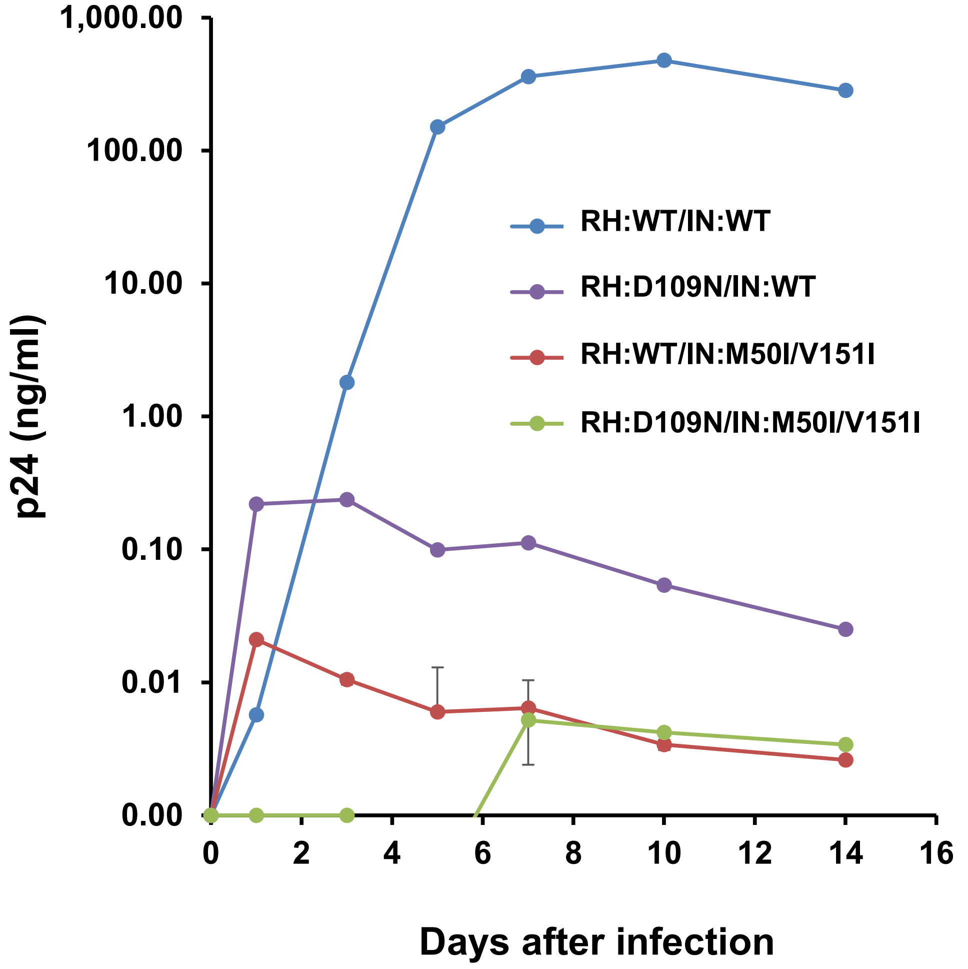

Figure S3. Impact of RH:D109N change on HIV replication in PHA-stimulated primary CD4(+) T cells. PHA-stimulated primary CD4(+) T cells from healthy donor were infected with 10 ng p24 amounts of HIV(WT) or variants containing mutations as described in Materials and Methods. The infected cells were cultured for 14 days with medium
changed every 3 to 4 days. HIV replication was monitored using a p24 antigen capture kit. Data presents as means ± standard deviations (SD) (n = 3).

Supplemental Figure S4

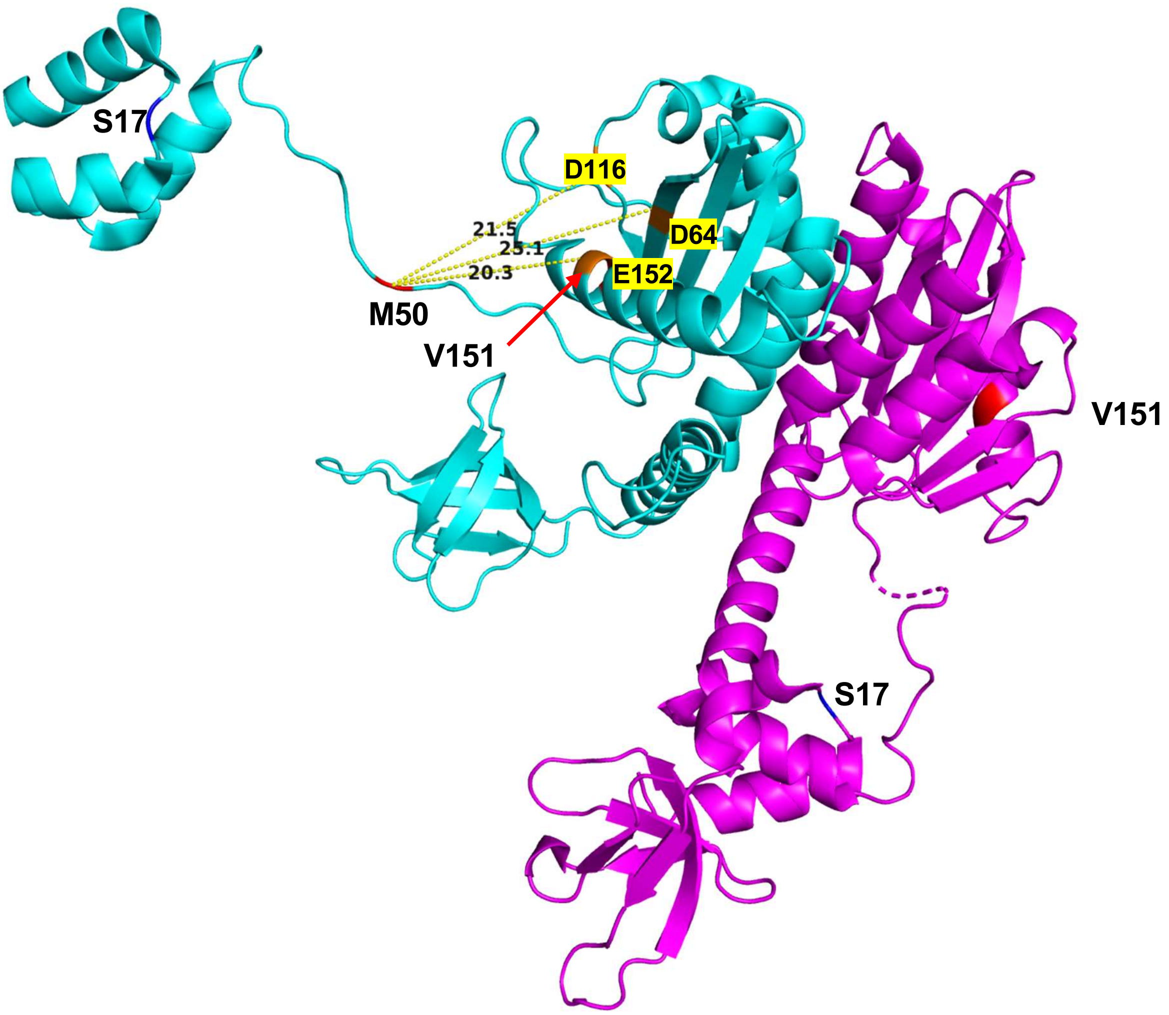

Supplemental Figure S4. 3D structure of IN (WT). The longest visible IN chains are available in 6U8Q. Chain A and B were extracted from 6U8Q and then aligned to IN dimer 5HOT with PyMOL. The common domains of 6U8Q and 5HOT are similar, indicating that Integrase Chain A and Chain B from 6u8q can be used as a basis of IN structure. Therefore, chains A and B of 6U8Q were used as a structural model in this study. First, we mutated the I151 to wild-type V151. Amino acid residues at the active site of IN, D64, D116, and E152 were colored orange. S17 is colored blue, and M50 and V151 are colored red. The distances between M50 and the active sites were calculated in PyMOL using the command: Distance chain A and i. 50 and n. CA, chain A, and i. (Coordinate of position B) and n. CA
