# Supplementary material for: The C-Terminal Domain of RNase H and the C-Terminus Amino Acid Residue Regulate Virus Release and Autoprocessing of a Defective HIV-1 Possessing M50I and V151I Changes in Integrase
Source: Viruses. 2022 Nov 30;14(12):2687. doi: 10.3390/v14122687 (PMC9788298; doi:10.3390/v14122687)
Supplement: Supplementary file 1 [file viruses-14-02687-s001.zip › Supplemental Tables S1-S5.pdf]

Supplemental Table S1. DNA sequence of primers for mutagnesis *

Primer names	5'-3'
RH:D109N.S	GGAGGAAATGAACAAGTAAATAAATTGGTCAGTGCTGGAATC
IN:D288G.S	GTGGCAAGTAGACAGGATGAGGGTTAACACATGGAAAAGATTAG
IN:D288N.S	GTGGCAAGTAGACAGGATGAGAATTAACACATGGAAAAGATTAG
IN:D288A.S	GTGGCAAGTAGACAGGATGAGGCTTAACACATGGAAAAGATTAG
IN:D288K.S	GTGGCAAGTAGACAGGATGAGAAATAACACATGGAAAAGATTAG
IN:D288E.S	GTGGCAAGTAGACAGGATGAGGAGTAACACATGGAAAAGATTAG
IN:D288W.S	GTGGCAAGTAGACAGGATGAGTGGTAACACATGGAAAAGATTAG
IN:D288Y.S	GTGGCAAGTAGACAGGATGAGTATTAACACATGGAAAAGATTAG
IN:Del288.S	GTGGCAAGTAGACAGGATGAGTAGTAACACATGGAAAAGATTAG
Delta RH:F	GGAATCAGGAAAGTACTATTTTTAGATGG
Delta RH:R	GGCTGCCCCATCTACATAGAAAGTTTCTGC
RH:1-51:R	GCAATTCATCTAGCTTTGCAGGATTCGGGATTA
RH:1-89:R	TTCCTTTTTTATTAACTGCTCTATTATTTGACTGAC
RH:1-98:R	GGAATTGGAGGAAATGAACAAG
Delta IN	GCTGGAATCAGGAAAGTACTATTTTAGATGGAATAGATAAGG
Delta CTD	GCTGGGGAAAGAATAGTAGACTAATAGCAACAGACATAC
Delta 14AA	CATCAGGGATTATGGATAAACAGATGGCAGGTG

* Sense strand sequence.

Supplemental Table S2. p values of combination of RH variants with IN:M50I/V151I/D288D

			IN:M50I/V151/D288D						
		WT	RH:1-120	RH:1-120/N79S	ΔRH	RH:1-51	RH:1-89	RH:1-98	RH:1-120/D109N
	WT								
IN:M50I/V151I/D288D	RH:1-120	****							
	RH:1-120/N79S	ns	**						
	ΔRH	ns	**	ns					
	RH:1-51	ns	***	ns	ns				
	RH:1-89	ns	**	ns	ns	ns			
	RH:1-98	**	ns	ns	ns	ns	ns		
	RH:1-120/D109N	ns	****	*	*	ns	*	**	

all statistical analyses were conducted by on way ANOVA using Prison 9.

*: $p<0.05$
**: $p<0.01$
***: $p<0.001$
****: $p<0.00001$

Supplemental Table S3. Population analysis of Codon 109 of RH in Database

LANL		Stanford		NIAID	
AA *	% **	AA	%	AA	%
D	99.62	D	100	D	100
F	0.02				
G	0.02				
H	0.02				
N	0.21				
?	0.11				

*: amino acid sequence at codon 109 of RH.

**: percentage of population of each amino acid in the database

***: Single letter code of amino acid; D: Aspartic acid, F: Phenylalanine, G: Glycine, H: Histidine, N: Asparagine, ?: uncharacterized aa

Supplemental Table S4. p values of combination of IN variants with IN:M50I/V151I *

			IN:M50I/V151/D288D										
		WT	ΔIN	D288D	ΔCTD	Δ14aa	D288G	D288N	D288K	D288A	D288E	D288Y	ΔD288D
	WT												
	ΔIN	*											
IN:M50I/V151I/D288D	D288D	****	*										
	ΔCTD	*	ns	*									
	Δ14aa	ns	*	****	ns								
	D288G	ns	ns	****	ns	ns							
	D288N	**	ns	*	ns	*	ns						
	D288K	ns	**	****	**	ns	ns	**					
	D288A	ns	ns	****	ns	ns	ns	ns	ns				
	D288E	ns	ns	****	ns	ns	ns	*	ns	ns			
	D288Y	ns	ns	****	ns	ns	ns	ns	ns	ns	ns		
	ΔD288D	ns	ns	****	ns	ns	ns	*	ns	ns	ns	ns	

*: Statistical analysis of virus release was conducted using one way ANOVA,

*: $p<0.05$, **:$P<0.01$, ***:$p<0.001$, ****:$p<0.0001$, ns: not significant, $p>0.05$

Supplemental Table S5. Poplulation analysis of amino acid residue at codon 288 of IN

	LANL		Sandford		NIAID	
AA at 288	Sequence*	% **	Sequence	%	Sequence	%
D **	4689	93.743	20826	97.85	495	96.12
E	5	0.1	4	0	0	0
G	5	0.1	16	0.1	2	0.39
N	301	6.018	208	1.9	18	3.5
S	2	0.04	2	0	0	0

*: Analyzed sequenced number in the database

**: Percentage of each amino acid residue in total analyzed sequences.

***: Single letter of amino acid; D: Asparatic acid, E: Glutamic Acid, G: Glycin, N: Asparagine, S: Serine.
